# Magnitude of Abdominal Wound Dehiscence and Associated Factors of Patients Who Underwent Abdominal Operation at St. Paul's Hospital Millennium Medical College, Addis Ababa, Ethiopia

**DOI:** 10.1155/2020/1379738

**Published:** 2020-02-23

**Authors:** Berhanetsehay Teklewold, Dut Pioth, Tadele Dana

**Affiliations:** ^1^Saint Paul Hospital Millennium Medical College, Department of Surgery, Addis Ababa, Ethiopia; ^2^Wolaita Sodo University College of Health Sciences and Medicine, Wolaita Sodo, Ethiopia

## Abstract

**Background:**

Abdominal wound dehiscence (AWD) is the separation of different layers of an abdominal wound before complete healing has taken place. It is a major cause of postoperative morbidity and mortality in sub-Saharan Africa including Ethiopia, and little is known about its prevalence and related factors in the study area.

**Objectives:**

The aim of this study is to assess the magnitude of abdominal wound dehiscence and related factors on patients operated at St. Paul's Hospital Millennium Medical College, Addis Ababa, Ethiopia.

**Methods:**

A hospital-based retrospective review of the chart was carried out by using the data covering three years (September 2014–September 2017) period. Data were collected from hospital medical records of sampled patients such as operation room logbooks and individual patient medical records. The collected data were checked for consistency, coded, and entered into SPSS version 20 for data processing and analysis. Descriptive analysis was conducted, and tables and graphs and summary statistics were used to depict data.

**Results:**

A total of 41 patients developed abdominal wound dehiscence from among 4137 patients who underwent abdominal laparotomy in the hospital. Among the patients, 51.2% were in the age range of 41 and above with mean age 29.8 (SD = 1.21) and 70.7% of them were male. Abdominal wound dehiscence was more common in emergency patients (90%) and vertical incision was the most common type of incision. Over half (58.5%) of the wound dehiscence occurred within 6–10 postoperative days. The majority (95.2%) of dehisced patients underwent relaparotomy for the management of the wound dehiscence, and 48.8% of them were treated with tension suture during the second operation of abdominal closure. Four of the patients (9.7%) died after the management of the second operation.

**Conclusion:**

The current study revealed that the overall magnitude of abdominal wound dehiscence in the study area was 0.99%. Most of the dehiscence has occurred in male patients, and older age groups were highly affected than the younger ones. Emergency admission is the most common form of admission identified in the study, and this signifies appropriate preoperative preparation of patients for an optimal outcome. However, regarding the management outcome, 9.8% of patients died in our study within the institution after the second operation which is the high mortality rate.

## 1. Background

Abdominal wound dehiscence (AWD) is a terminology that is commonly used to explain the separation of different layers of an abdominal wound before complete healing has taken place. Other terms used are acute laparotomy wound failure and burst abdomen [1]. It usually occurs when a wound fails to achieve the required strength to withstand stresses placed upon it [2, 3].

Abdominal wound dehiscence is one of the most dreaded life-threatening complications owing to the associated rapid onset of often irreversible pathological sequel. It is a major cause of postoperative morbidity and mortality in sub-Saharan Africa including Ethiopia [4–6]. Unlike the encouraging outcome recorded in more developed countries, associated mortality is very high in many developing countries due to infective complications and lack of adequate facilities [1, 7].

The magnitude of wound dehiscence varies from hospital to hospital worldwide. It is recorded to be 1–3% in most hospitals with an impact of mortality rate as high as 45% [1, 8, 9]. Different combinations of factors are identified as risk factors in several studies [9–11] and are mostly classified as local and general factors. So, early identification of these factors and doing simple routine laboratory investigations may help in reducing the occurrence of wound dehiscence [9–15]. Patients undergoing emergency surgery are more at risk to develop abdominal wound dehiscence as compared to the patient undergoing elective surgery [16], and different studies have shown that its incidence is common in older age groups [17–21].

Nearly half the adverse events following postoperative complications are considered to be preventable [22] by doing the appropriate surgical technique and wound care with sterile techniques [23] and also by improving the nutritional status of the patient, strict aseptic precautions, and improving patient's respiratory pathology to avoid postoperative cough [24].

This study is aimed to assess the magnitude of abdominal wound dehiscence and also to describe patient and clinical factors associated with it in the study area.

## 2. Methods

### 2.1. Study Design and Period

An institution-based cross-sectional study was conducted from May to June 2018.

### 2.2. Study Area

The study was conducted at Saint Paul Hospital Millennium Medical College (SPHMMC), a tertiary teaching hospital, which is located in the Northern part of Addis Ababa, capital of Ethiopia. St. Paul's hospital is the second-largest hospital in Addis Ababa which serves as a referral centre for patients from Addis Ababa and all over the country. The hospital serves as a teaching and treatment centre in surgery, internal medicine, gynaecology and obstetrics, paediatrics and child health, maxillofacial surgery, psychiatry, ophthalmology, pathology, and radiology. The department of surgery is one of the major departments, divided into outpatient, inpatient department, and minor and major operating theatres. The inpatient department services include general surgery, urologic surgery, neurosurgery, paediatric surgery, hepatobiliary, renal transplantation, laparoscopic surgery, and vascular surgery.

### 2.3. Source and Study Population

All patients who underwent abdominal surgery or laparotomy in the hospital were the source population, and all patients who underwent abdominal operation from September 2014 to September 2017 at SPHMMC, department of surgery were the study population.

### 2.4. Inclusion and Exclusion Criteria

- All paediatrics and adults of either sex who underwent abdominal laparotomy
- All patients with complete records
- All patients who have developed wound dehiscence after second surgery or third surgery were excluded.

### 2.5. Sample Size Determination and Sampling Technique

All patients who underwent abdominal surgery registered from September 2014 to September 2017 at St. Paul's Hospital Millennium Medical College, Department of Surgery, were taken as a sample.

### 2.6. Data Collection Materials

A structured checklist was used to collect the data on sociodemographic characteristics, clinical factors, and information about the outcome of management by reviewing the charts of the patients.

### 2.7. Data Processing and Analysis

All questionnaires were checked for completeness and consistency of responses manually. To assure the quality of the data, check-up for completeness and consistency of the data was made by the investigator. After editing, data were entered into SPSS versions 20 for analysis. Descriptive statistics (frequencies and percentages) were used to explain the study participant to study variables. Texts, tables, and charts were used to display results. A frequency and crosstab descriptive analysis was used.

### 2.8. Ethical Consideration

Ethical clearance was obtained from the ethical review board of SPHMMC. To ensure the confidentiality of respondents, their names were left out on the questionnaire, and all the collected data were kept only for this research work.

## 3. Result

### 3.1. Sociodemographic Characteristics of the Patients with Wound Dehiscence

A total of 41 (0.99%) patients developed abdominal wound dehiscence from September 2014 to September 2017 among 4137 patients who underwent abdominal surgery at the SPHMMC, Department of Surgery. The mean age of patients was 29.8 (SD = 1.21) years with 1 year and 80 years being the lowest and oldest age, respectively. Among the patients, the majority 21 (51.2%) were in the age range of 41 and above and 29 of the patients (70.7%) were male (see Table 1).

### 3.2. Clinical Characteristics of the Patients

Regarding clinical characteristics of the patients, the majority 37 (90.2%) underwent emergency surgery, and 21 (51.5%) of them had no comorbid illness, but anemia was a frequent preoperative comorbidity with a frequency of 24% of those with clinical comorbidities. Twenty-eight (68.3%) of them were operated for acute abdomen secondary to bowel obstruction (both small and large bowel obstruction). Thirty-six (87.8%) of the incisions were vertical midline incision and 24 (58.5%) of them developed wound dehiscence within 6–10th postoperative days. Regarding the management-related issues, 39 (95.2%) of them underwent relaparotomy for the management of the wound dehiscence, and 20 (48.8%) of them were treated with tension suture, and only 22% were treated with layered suture during the second operation of abdominal closure (see Table 2).

### 3.3. Management Outcome among Patients Who Developed Abdominal Wound Dehiscence

The current study showed that 37 (90.2%) of the patients were alive and discharged home after the second management; however, 4 (9.8%) of patients died within the institution after the second surgical management.

## 4. Discussion

The current study revealed that the overall magnitude of abdominal wound dehiscence in the study area is 0.99%. This finding is similar to the studies carried out in New York, USA 1% [25], but it was slightly higher than the study carried out in Mesologgi General Hospital 0.43% [7]. On the contrary, the current study finding is lower than the studies done in Siddhartha Medical College, in Pakistan Institute of Medical Sciences, Islamabad, 5.9% [12], and in RNT Medical College, Udaipur, India, 5.38% [10]. The reason might be due to the difference in the study population that those who were above 70 years old patients with the mean age was 69.5 years were in the sample in Mesologgi General Hospital [7].

Regarding clinical factors, those who were operated for emergency (90.24%), those operated for acute abdomen secondary to bowel obstruction during the 1st surgery (68.3%), and those who underwent vertical midline incision (87.80%) were more likely to develop abdominal wound dehiscence than the other groups of patients. The reason for this might be because these procedures are life-saving procedures, and patients are rushed for operation with short times for stabilization and adequate resuscitation which hugely affect the operative outcome; also, keeping sterility of procedures is also poor during emergency hours as compared to elective hours. The other factor that might be associated with this is the suturing technique and also use of suturing material used in emergency conditions. In elective surgeries, it is a standard practice to close midline vertical incisions with slowly absorbable sutures and at small steps with continuous technique in a ratio of 4 : 1. Different studies have also shown that the same technique used during emergency condition has significantly dropped the rate of dehiscence [26]. In our studies, though practice of technique and type of suturing material used were not described, 87% of incisions were vertical, and 90% of patients had emergency condition; therefore, it is useful to audit routine practices and adopt best practices identified in other studies to reduce rate of dehiscence. Greater than 70% of patients in our study were treated by the mass and tension suture technique during the second operation. According to experimental studies on pigs, mass closure technique showed more wound separation when compared with layered [27] but systematic reviews by Ceydeli et al. concluded mass closure to be used as a standard [28]. To date, the recommendation on use of ideal tension on abdominal closure remains unknown.

Anaemia was identified as the common comorbidity (50%) among those with comorbidity. Anaemia implies low oxygen supply to tissue, and this, in turn, affects tissue healing and resistance to infection decreases too. This finding was similar to the studies conducted in Osmania General Hospital that showed 63.63% of patients had anaemia as comorbidity [20].

Concerning the management outcome of the second surgery, the current study showed that 9.8% of patients died within the institution after the second operation, which is lower than the studies conducted in Mesologgi General Hospital 20% [7], 45% in Pakistan [11], and 39.3% in Wiad Lek [6]. The reason might be due to the different sample sizes and the difference in the sociodemographic characteristics of the patients.

Regarding factors related to management outcome, those patients who were operated for emergency condition (10.81%), who had pulmonary disease as a clinical comorbid illness (50%), those who underwent vertical midline incision (11.11%), those who had relaparotomy during the 2nd surgery (10.25%), and those who had tension suture of abdominal closure during 2nd surgery (15%) had poor management outcome (dead). The current study finding is supported by the other study finding like the study carried out in Osmania General Hospital which showed 72.72% of patients with emergency laparotomies, 51.51% of patients with peritonitis, 63.63% of patients with anaemia, and 51.51% of patients with respiratory infections [20]; in India, patients with complicated appendicitis, anaemia (56%), and patients treated as emergency surgeries (92%) [2] were affected.

## 5. Conclusion and Recommendation

In our study, the fact that dehiscence commonly occurred at emergency hours and also on elderly patients shows that these circumstances need special attention for preoperative care to minimize the occurrence of this disastrous complication. Strict follow-up of sterility techniques and also auditing use of type of sutures are mandatory to decrease the incidence. Surgeon-related factors and also techniques of abdominal closure used during emergency condition should also be sought carefully to identify preventable causes. In most setups, emergency procedures are handled by residents and even elective cases; though attended by consultants, most of the time skin and abdominal closures are left to residents to close. Therefore, strict monitoring and adherence to surgical principles are very important.

## Figures and Tables

**Table 1:** Descriptions of sociodemographic factors among patients who developed abdominal wound dehiscence during September 2014 to September 2017 at SPHMMC, Addis Ababa, Ethiopia (*n* = 41).

Variables		Frequency	Percent (%)
Age	Below 41 years	20	48.8
	41 and above years	21	51.2

Sex	Male	29	70.7%
	Female	12	29.3%

**Table 2:** Description of clinical factors among patients who developed abdominal wound dehiscence during September 2014 to September 2017 at SPHMMC, Addis Ababa, Ethiopia (*n* = 41).

Variables		Frequency	Percent (%)
Urgency of surgery	Elective	4	9.8
	Emergency	37	90.2

Comorbid clinical illness	Anemia	10	24
	Malnutrition	4	9.8
	Pulmonary diseases	2	4.9
	Malignancy	4	9.8
	No comorbid illness	21	51.5

Indication for surgery	Acute abdomen secondary to penetrating abdominal injury	4	9.8
	Acute abdomen secondary to bowel obstruction	28	68.3
	Acute abdomen secondary to appendicular abscess	2	4.9
	Peptic ulcer disease perforation	3	7.3

Type of incision	Vertical midline	36	87.8
	Transverse right subcostal	4	9.8
	Transverse right lower abdominal	1	2.4

Postoperative day of wound dehiscence	0–5	13	31.7
	6–10	24	58.5
	11–15	4	9.8

Mode of management	Relaparotomy	39	95.2
	Conservative	2	4.8

Abdominal closure in the 2^nd^ operation	Mass closure	10	24.4
	Tension suture	20	48.8
	Layered closure	9	22
	Conservative management	2	4.8

## Data Availability

The data used to support the findings of this study are available from the corresponding author upon request.
